# Preparation and Density Functional Theory Studies of Aluminosilicate-Based Ceramic Solidified Products for Sr Immobilization

**DOI:** 10.3390/toxics11100850

**Published:** 2023-10-11

**Authors:** Yan Wu, Hongji Sang, Jiawei Zheng, Shuyi Yang, Zhengcheng Gu, Hao Wu, Yuezhou Wei

**Affiliations:** School of Nuclear Science and Engineering, Shanghai Jiao Tong University, Shanghai 200240, China

**Keywords:** solidification, DFT calculation, Sr, allophane

## Abstract

Strontium is a common radionuclide in radioactive waste, and its release into the environment can cause enormous damage to the ecosystem environment. In this study, the natural mineral allophane was selected as the substrate to prepare solidified ceramic products by cold pressing/sintering to solve the problem of the final disposal of radioactive strontium. Ceramic solidified products with various crystal structures were successfully prepared, and the microscopic morphology and energy-dispersive spectroscopy images of the samples showed a uniform distribution of Sr in the solidified products. Sr_2_Al_2_SiO_7_ and SrAl_2_Si_2_O_8_, which can stably solidify strontium, were formed in the solidified products, and the structural characteristics and stability of the above-mentioned substances were analyzed from the perspective of quantum chemical calculations using density functional theory. The calculation results showed that the overall deformation resistance of Sr_2_Al_2_SiO_7_ was higher than that of SrAl_2_Si_2_O_8_. Considering the isomorphic substitution effect of CaO impurities, we inferred that a mixed-crystalline structure of Ca_2−x_Sr_x_Al_2_SiO_7_ may be present in the solidified products.

## 1. Introduction

Nuclear energy is a crucial high-quality energy source, and its development provides a new impetus for the progress of human society. However, nuclear energy production inevitably generates hazardous radioactive waste, posing substantial environmental and biological risks. Thus, it must be treated safely and effectively [1]. The radionuclide ^90^Sr, characterized by radiotoxicity, high heat generation, and a long half-life of 28.8 years, is a β-emitter generated by uranium and plutonium fission reactions in nuclear reactors [2,3,4]. As an alkaline-earth cation with chemical properties similar to calcium, ^90^Sr easily accumulates in human bones [5,6]. ^90^Y, a daughter nuclide of ^90^Sr, produces high-energy β particles that can damage the bone marrow. Therefore, considering environmental protection and human health, the safe disposal of ^90^Sr from radioactive waste is valuable to a great extent [7].

Stabilization and solidification are considered the most promising technologies for radionuclide management [8,9]. In addition to traditional glass and cement solidification, ^90^Sr solidification primarily focuses on geopolymer and ceramic solidifications. Geopolymers are amorphous inorganic binder materials that are usually composed of SiO_4_ and AlO_4_ tetrahedra and can be prepared from solid wastes, such as fly ash, metakaolin, and slags via alkali activation at ambient temperature [10,11,12]. These materials can be considered precursors to zeolites with different affinities toward various ions. Notably, the Si/Al ratio is the controlling factor affecting the adsorption ability of geopolymers [13,14,15,16]. In the field of radioactive waste immobilization, geopolymers that outperform Portland cement can be prepared by changing the precursor materials and activators [15]. The absorbed waste elements can be further converted into stable ceramic-phase components, thereby increasing the difficulty of ion leaching by strengthening chemical bonds. The ceramic solidification approach has received extensive research attention due to its excellent chemical stability, radiation stability, and leaching resistance [17]. At the atomic scale, radioactive elements can enter the lattice structure of ceramic matrix materials, thereby forming a safer immobilization barrier than in glass [18]. At present, various ceramic materials, such as phosphates, aluminosilicates, titanates, zirconates, and vanadates [7,17,18,19,20], have been extensively studied as matrices for immobilizing radioactive wastes through methods, such as cold pressing/sintering, microwave sintering, and spark plasma sintering [21,22,23,24].

As inorganic polymers, aluminosilicates have layered and skeletal crystal structures that endow them with high specific surface areas. These materials can achieve the selective adsorption of multiple ions through a molecular sieve mechanism; that is, certain ion sizes can enter the cavities, pores, and channels formed by the aluminosilicate framework. Allophane is a short-range order aluminosilicate and an affordable material for obtaining solid-state matrices based on aluminosilicate ceramics. It is primarily found in volcanic ash soil, and the basic structure of its skeleton comprises Al(OH)_3_ and SiO_4_ [25]. The special frame structure of allophane confers good radiation resistance stability and the ability to capture gases [26]. Owing to these advantages, it is one of the most promising materials for metal-ion removal and immobilization. Our group previously treated a mixture of allophane and cesium secondary waste at high temperatures, resulting in the breakdown of the allophane framework and the formation of stable crystalline phases that encapsulate cesium [27,28]. Excellent leaching resistance and mechanical properties are the main characteristics of the prepared solidified ceramic bodies. However, systematic studies on the application of allophane for the immobilization of highly radioactive ^90^Sr have rarely been reported. 

The use of density functional theory (DFT) to study the binding behavior of metal ions in lattice-defect structures and lattices can enable a deeper understanding of the solidification structures and bonding mechanisms at the molecular level. The lattice parameters, structural stability, mechanical properties, thermo-physical properties, and electronic structure of different materials have been extensively studied through DFT experiments to evaluate their comprehensive properties [29,30,31,32,33,34]. However, few studies have been conducted on the immobilization mechanism of Sr in high-temperature ceramics. Considering the stability and advantages of capturing Sr in ceramic matrices, it is important to explore the immobilization mechanism of solidified ceramic products at the molecular level.

In this study, the natural mineral allophane was used to synthesize solidified Sr ceramic products through cold pressing/sintering. The performance and mechanism of the allophane on Sr were explored, and the structural characteristics and stability of the sintered products were analyzed from the perspective of quantum chemical calculations using DFT.

## 2. Materials and Methods

### 2.1. Materials

**Figure 1:**
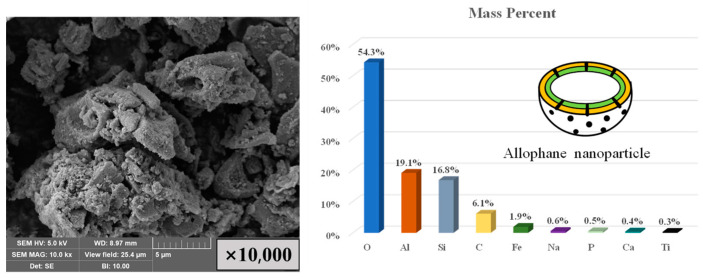
SEM images (**left**) and principal compositions of natural allophanes (**right**).

Nonradioactive ^87^Sr was used in all samples instead of radioactive ^90^Sr. Analytical-reagent-grade strontium nitrate (Sr(NO_3_)_2_) was purchased from Sinopharm Chemical Reagent Co. Ltd., China. Allophane (1–2SiO_2_·Al_2_O_3_·5–6H_2_O), with an average particle size of 5.9 µm, was obtained from Hattori Company, Ltd., Japan. The smaller particle size ensured a larger specific surface area, which was conducive to improving the adsorption capacity of metal ions. The surface morphology and main components of the allophane used as matrices are shown in Figure 1.

### 2.2. Preparation of Solidified Products

The solidified product was prepared as follows: Allophane and Sr(NO_3_)_2_ were uniformly mixed in various mass ratios. Then, the uniform mixture was ground into a fine powder and molded into a disk via the cold pressing method using hydraulic pressure tablets under 4 kN with a holding time of 6 min. The resulting 10 mm diameter molded discs were placed in a muffle furnace for sintering at specific temperatures and a heating rate of 10 °C/min for 1 h.

### 2.3. Characterization

Scanning electron microscopy and energy-dispersive spectrometry (SEM and EDS, Mira 3, TESCAN ORSAY HOLDING a.s., Brno, Czech Republic) were used to measure the surface morphology and elemental distribution of the solidified products. The mass fractions of each element in the samples before and after sintering were determined using an X-ray fluorescence spectrometer (XRF-1800, Shimadzu Corporation, Kyoto, Japan). The crystal structures of the samples were examined using powder X-ray diffraction (XRD) spectroscopy (D8 ADVANCED DAVINCI, Bruker Corporation, Karlsruhe, Germany).

### 2.4. DFT Calculation

DFT is a classical quantum mechanical modeling method for calculating and predicting the electronic properties of crystal structures with high precision. It has been used extensively in computational research on ceramic materials. This method calculates the energy of the system as a function of electron density without solving the complex many-body Schrodinger equation, thereby significantly simplifying the calculation. In this study, DFT calculations were performed to analyze the crystal structure and mechanical properties of the solidified ceramic products. The gain and loss of electrons and chemical bond types of the Sr atoms were also studied to analyze the chemical stability of the solidified products. All calculations in this study were carried out based on the DFT method using the Vienna ab initio simulation package (VASP). Electron exchange and related energy were computed using the projector augmented wave (PAW) method with the Perdew–Burke–Ernzerhof generalized gradient approximation (PBE-GGA) function [35,36]. The Brillouin zone sampling was performed using a Gamma grid. The cut-off energy was set to 400 eV, as confirmed by the convergence test, and the force convergence of the structural optimization was set to be lower than 0.02 eV/Å.

## 3. Results and Discussion

### 3.1. Characterization and Self-Sintering Behavior of Allophane

**Figure 2:**
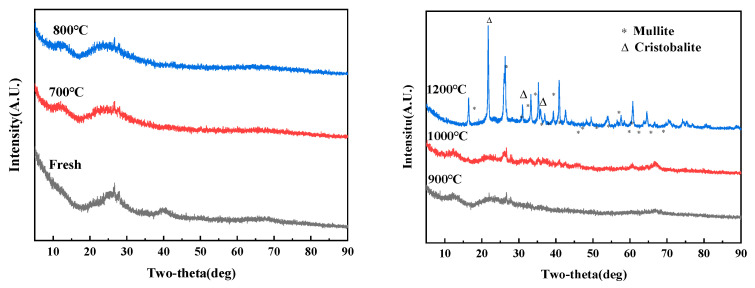
XRD patterns of allophanes sintered at different temperatures.

Self-sintering experiments were performed on the allophanes at different temperatures. The XRD patterns of allophanes with different sintering temperatures are shown in Figure 2. No obvious diffraction peaks were observed in the XRD patterns of fresh allophane, indicating that allophane was an amorphous substance. Two weak diffraction peaks were observed at 26.6° and 28°, which may be attributed to the spherical shell structure of allophane, comprising silica and alumina, characterized by short-range ordering. When the sintering temperature was lower than 1000 °C, wide and diffuse peaks were observed in the XRD pattern, suggesting no mineral-phase transformation or crystal-phase substance formation under these experimental conditions. After sintering at 1200 °C, the main crystal phases in the self-sintering products of allophane were primarily transformed into two phases, namely, mullite (Al_6_Si_2_O_13_) and cristobalite (SiO_2_). The increase in the sintering temperature may have caused the Si–O–Si and Si–O–Al bonds to break and the allophane framework to collapse. The decomposition products Al_2_O_3_ and SiO_2_ with high chemical reaction activity also recrystallize at high temperatures to yield a mullite crystal phase [26,37]. The content of the cristobalite crystal phase was relatively low, and the cristobalite phase may be attributed to the high-temperature calcination of excessive SiO_2_ [38]. Moreover, the volume reduction rate of the sintered solidified body increased from 20.6% to 52.2% with increased sintering temperature from 700 °C to 1000 °C. This phenomenon has benefited waste minimization and improved economic efficiency. Hence, the formation of stable crystalline phases and good volume reduction rates indicate that allophane has excellent self-sintering properties, making it a potential matrix material for the solidification of radionuclide ceramics.

### 3.2. Sr Immobilization Ratio and Solidification

**Figure 3:**
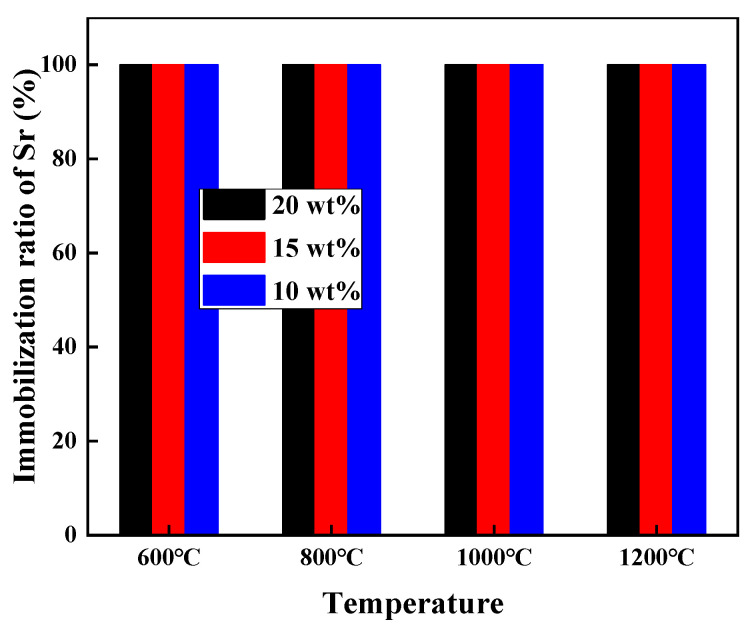
Sr immobilization ratios at different sintering temperatures.

Figure 3 shows the Sr immobilization ratios obtained via XRF for various mixtures of Sr(NO_3_)_2_–allophane sintered at 600–1200 °C, with a gradual increase in Sr content from 10wt% to 20 wt%, the immobilization ratio of Sr remained at approximately 100% at all sintering temperatures. This finding indicates that allophane can be effectively used for the immobilization treatment of Sr.

**Figure 4:**
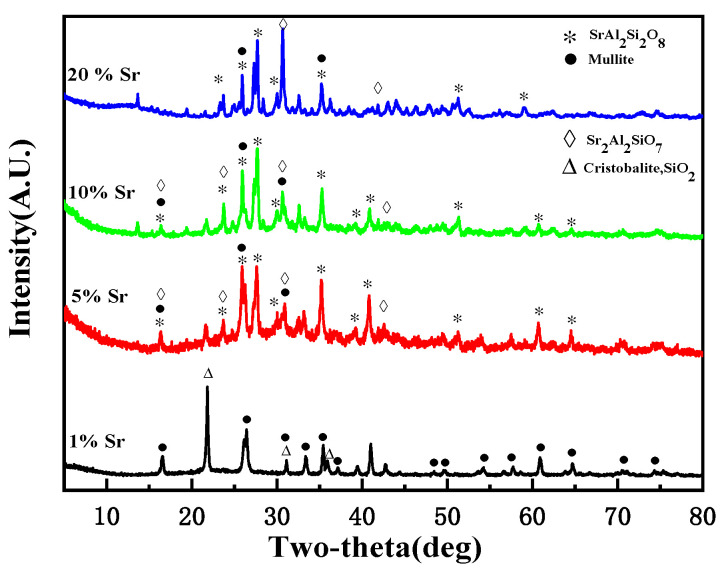
XRD patterns of solidified products at 1200 °C.

XRD was used to study the crystal phases of the sintered products and to understand the immobilization mechanism of Sr. Figure 4 shows the XRD patterns of various mixtures of Sr(NO_3_)_2_–allophane sintered at 1200 °C. The crystal structure of the solidified products changed after sintering. When the Sr content was 1 wt%, no obvious crystal phase of Sr was detected in the solidified product; it had primarily mullite and cristobalite crystal phases. Thus, the self-sintering behavior of allophane dominated the immobilization when the Sr content was low. When the Sr content ranged from 5 wt% to 30 wt%, in addition to the mullite phase, strontium feldspar (SrAl_2_Si_2_O_8_) and strontium aluminum silicate oxide (Sr_2_Al_2_SiO_7_) crystal phases that can stably solidify Sr also formed in the solidified product. With increasing Sr content in the solidified product, the proportion of the mullite phase gradually decreased and that of Sr_2_Al_2_SiO_7_ gradually increased; however, the proportion of SrAl_2_Si_2_O_8_ initially increased and then decreased. This finding may be related to the value of Si/Al in the solidified product. The deflection of the diffraction peaks can also be observed in the solidified products with different compositions. According to the Scherrer formula, the decreased interplanar spacing led to an increased diffraction angle, indicating that the crystal-phase structure of the solidification products changed to a certain extent.

**Figure 5:**
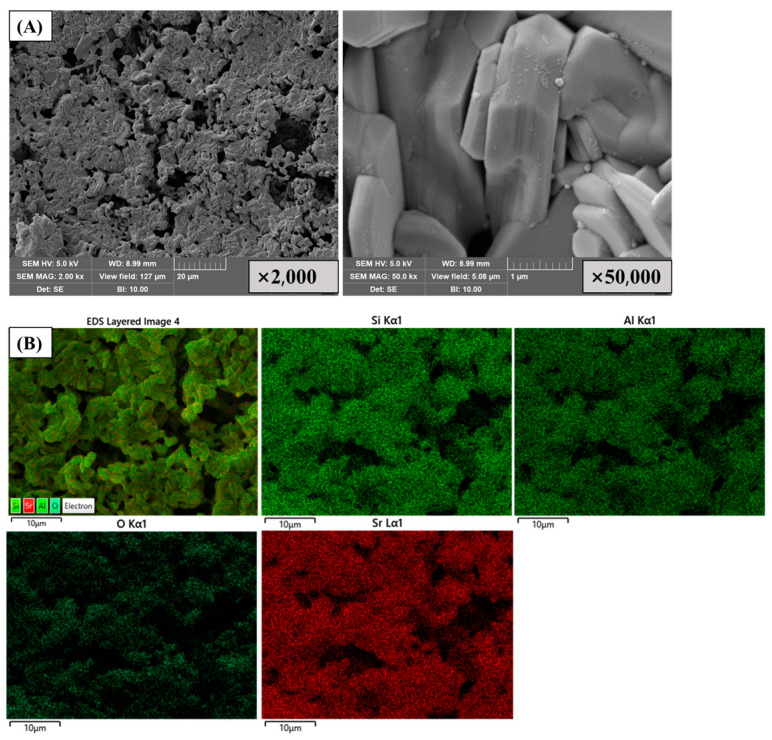
(**A**) SEM images and (**B**) EDS mapping of solidified product.

The surface morphologies and main elemental compositions of the solidified products after sintering at 1200 °C are shown in Figure 5. The surface of the solidified product appeared to melt and transform into a ceramic phase, and a non-uniform porous surface was formed. Si, Al, and Sr were detected and uniformly distributed on the surface of the solidified product, indicating that well-crystallized phases containing Sr were produced, which is consistent with the XRD results.

### 3.3. Calculation of Elastic Properties of Solidified Product

**Figure 6:**
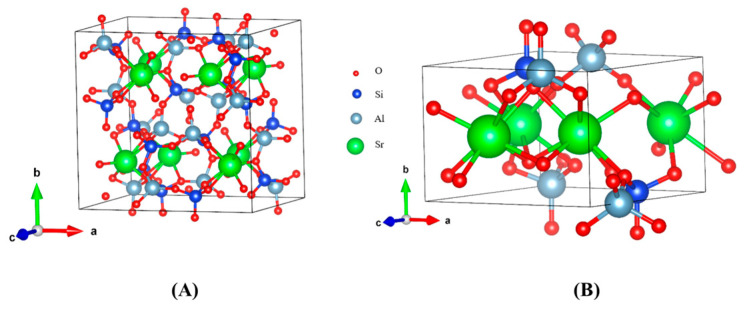
Structures of (**A**) SrAl_2_Si_2_O_8_ and (**B**) Sr_2_Al_2_SiO_7_ after optimization.

Sr was primarily immobilized in the solidified product in the form of SrAl_2_Si_2_O_8_ and Sr_2_Al_2_SiO_7_. Therefore, the Young’s modulus, shear modulus, and other parameters of the two phases were simulated and calculated using DFT to analyze the change in mechanical properties of the solidified product with increased Sr content. The crystal-structure parameters of SrAl_2_Si_2_O_8_ and Sr_2_Al_2_SiO_7_ after optimization are listed in Table 1, and the structures of SrAl_2_Si_2_O_8_ and Sr_2_Al_2_SiO_7_ are shown in Figure 6. The calculated elastic-tensor parameters of the two crystals after optimizing the cell structure are listed in Table 2.

In general, the hardness of a material increases with an increase in Young’s modulus (*E*). Conversely, a smaller shear modulus (*G*) corresponds to more easily formed dislocation slips, causing the material to become ductile [36]. The bulk modulus (*B*) of polycrystalline crystals was used to measure the relationship between the bulk strain and the average stress of the crystal. The bulk modulus decreased with an increase in the crystal volume. The bulk modulus, shear modulus, Young’s modulus, and Poisson’s ratio ν were evaluated using the Voigt–Reuss–Hill approximation (Equations (1)–(3)) [36].

(1) $$\begin{array}{cc} B_{V}=\frac{1}{9}\left(C_{11}+C_{22}+C_{33}\right)+\frac{2}{9}\left(C_{12}+C_{13}+C_{23}\right) & \\ B_{R}=\frac{1}{\left(S_{11}+S_{22}+S_{33}\right)+2\left(S_{12}+S_{13}+S_{23}\right)} & \end{array}$$

$$G_{V}=\frac{1}{15}\left(C_{11}+C_{22}+C_{33}-C_{12}-C_{13}-C_{23}\right)+\frac{1}{5}\left(C_{44}+C_{55}+C_{66}\right)$$

(2) $$G_{R}=\frac{15}{4\left(S_{11}+S_{22}+S_{33}\right)-4\left(S_{12}+S_{13}+S_{23}\right)+3\left(S_{44}+S_{55}+S_{66}\right)}$$

$$B=\frac{B_{V}+B_{R}}{2},G=\frac{G_{V}+G_{R}}{2},$$

(3) $$E=\frac{9BG}{3B+G},\nu =\frac{3B-E}{6B}$$

where *C*_ij_ and *S*_ij_ represent the elastic constant and elastic compliance, respectively.

The anisotropy indices of the bulk and shear moduli can be expressed using the following formula [39]:

(4) $$A_{B}=\frac{B_{V}-B_{R}}{B_{V}+B_{R}},A_{G}=\frac{G_{V}-G_{R}}{G_{V}+G_{R}}$$

**Figure 7:**
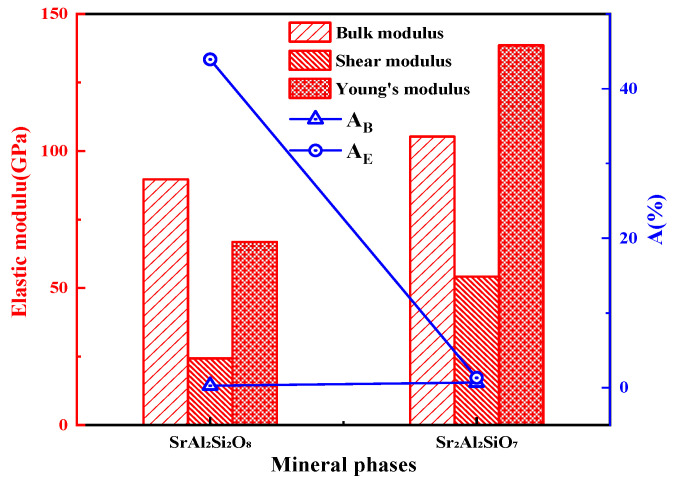
Elastic modulus and the anisotropy of SrAl_2_Si_2_O_8_ and Sr_2_Al_2_SiO_7_.

The calculated bulk, shear, and Young’s moduli of the SrAl_2_Si_2_O_8_ and Sr_2_Al_2_SiO_7_ polycrystals are shown in Figure 7. The results showed that the overall anti-deformation ability of Sr_2_Al_2_SiO_7_ was better than that of SrAl_2_Si_2_O_8_. Moreover, a larger value of *A* corresponds to greater anisotropy of the system, whereas when *A* is equal to zero, it is completely isotropic [36]. The anisotropy percentages of the bulk and shear moduli of Sr_2_Al_2_SiO_7_ are close to 0, further indicating that this crystalline phase is isotropic. By contrast, SrAl_2_Si_2_O_8_ showed obvious anisotropy.

**Figure 8:**
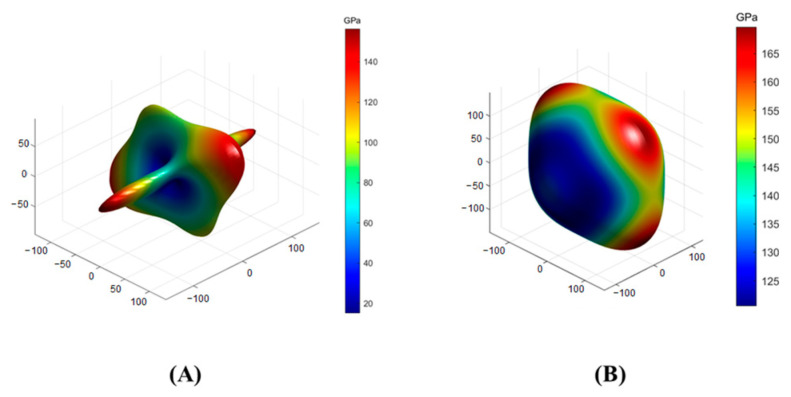
Anisotropy of the Young’s modulus in (**A**) SrAl_2_Si_2_O_8_ and (**B**) Sr_2_Al_2_SiO_7_.

**Figure 9:**
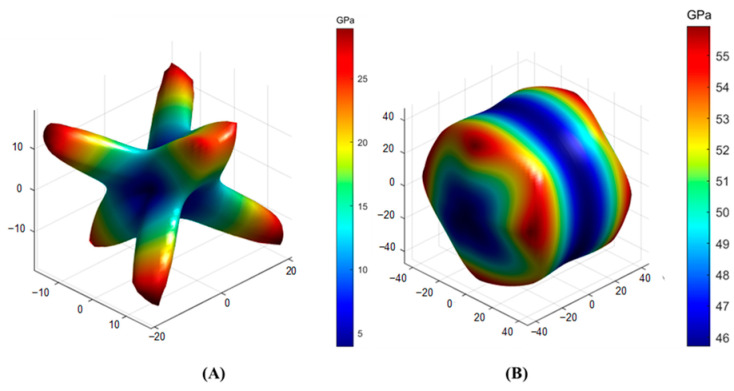
Anisotropy of the shear modulus in (**A**) SrAl_2_Si_2_O_8_ and (**B**) Sr_2_Al_2_SiO_7_.

To observe the anisotropy property more clearly, the three-dimensional distributions of Young’s modulus and shear modulus were obtained through further calculation [40], and the results are displayed in Figure 8 and Figure 9, respectively. The closer the three-dimensional figure was to a spherical shape, the more isotropic the system. Obviously, SrAl_2_Si_2_O_8_ was more anisotropic than Sr_2_Al_2_SiO_7_ in terms of Young’s modulus and shear modulus.

### 3.4. Simulation and Calculation of Sr/Ca Mixed-Crystal Structures

**Figure 10:**
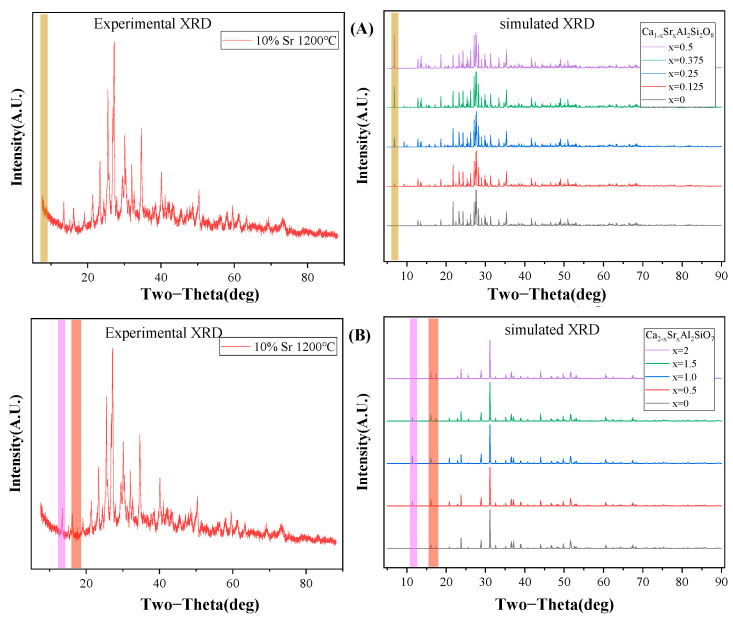
Comparison of experimental and simulated XRD patterns: (**A**) SrAl_2_Si_2_O_8_ and Ca_1−x_Sr_x_Al_2_Si_2_O_8_, and (**B**) Sr_2_Al_2_SiO_7_ and Ca_2−x_Sr_x_Al_2_SiO_7_. (Green column: 2θ = 6.8°; pink column: 2θ = 11.38°; red column: 2θ = 16.12° and 17.38°).

Given that allophane contains some CaO impurities, considering the isomorphism substitution, a Sr-Ca mixed-crystal phase may have existed in the solidified product after sintering. According to the doping levels of different Ca atoms, the XRD patterns of Ca_1−x_Sr_x_Al_2_Si_2_O_8_ and Ca_2−x_Sr_x_Al_2_SiO_7_ after optimization are shown in Figure 10A,B. As shown in Figure 10A, for the Sr/Ca mixed-crystal structure of SrAl_2_Si_2_O_8_ (simulated), an obvious diffraction peak was observed at 2θ = 6.8° with a change in the Ca/Sr ratio. The intensity of the diffraction peak increased as the Ca/Sr approached one. The XRD pattern of the experimental test revealed no obvious diffraction peak at 2θ = 6.8°. Therefore, we speculated that no mixed-crystal formation of Ca_1−x_Sr_x_Al_2_Si_2_O_8_ occurred.

**Figure 11:**
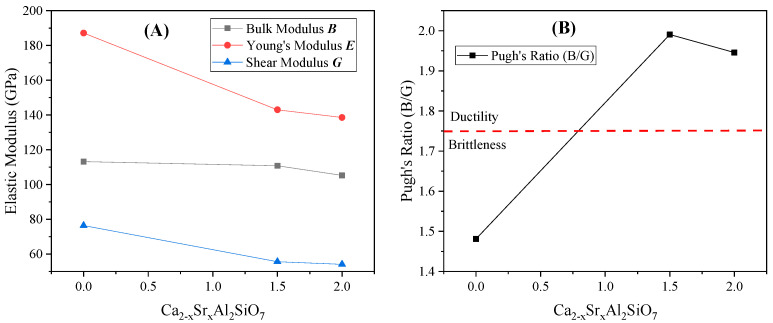
(**A**) Elastic modulus and (**B**) Pugh’s ratio of Ca_2−x_Sr_x_Al_2_SiO_7_.

As shown in Figure 10B, unlike SrAl_2_Si_2_O_8_, obvious diffraction peaks were observed at 2θ = 11.38°, 16.12°, and 17.38° in the XRD patterns of Sr_2_Al_2_SiO_7_ (experimental) and Ca_2−x_Sr_x_Al_2_SiO_7_ (simulated). This finding suggested that a mixed-crystal structure of Ca_2−x_Sr_x_Al_2_SiO_7_ formed in the sintered solidified product. The mechanical properties of the Ca_2−x_Sr_x_Al_2_SiO_7_ mixed crystals that appeared in the experiment were calculated, and the elastic moduli are shown in Figure 11A. With increased Sr content, the bulk modulus, Young’s modulus, and shear modulus of the polycrystal showed a downward trend, indicating that adding Sr caused a deterioration in mechanical properties. According to the Pugh criterion, the ratio of B/G was generally 1.75 as the boundary ratio between ductility and brittleness. These results confirmed that the brittle crystal transformed into a ductile one with increased Sr content, as shown in Figure 11B.

### 3.5. Analysis of Electron Localization Function (ELF)

**Figure 12:**
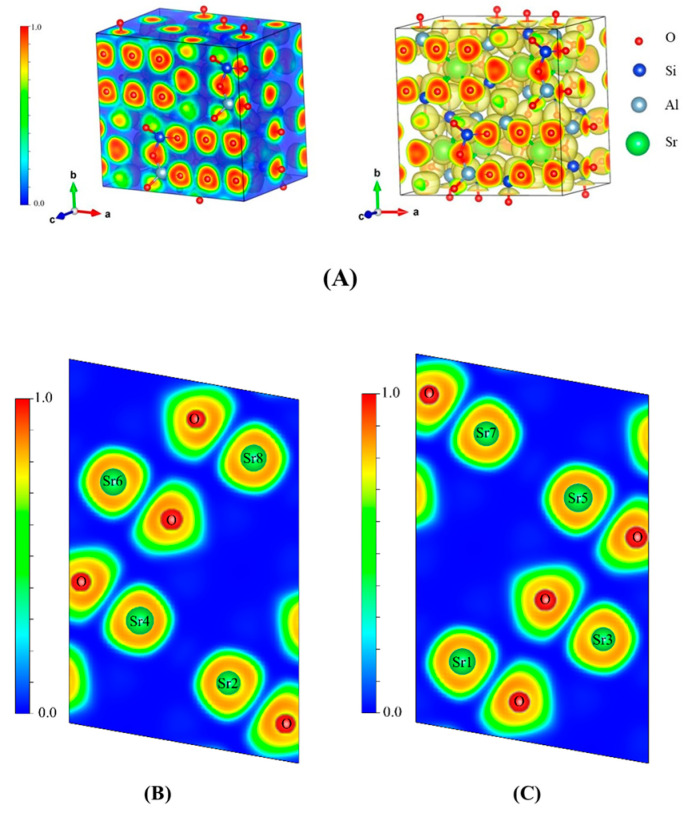
(**A**) Three-dimensional ELF diagrams of SrAl_2_Si_2_O_8_ (**B**,**C**) are two-dimensional electron localization diagrams of SrAl_2_Si_2_O_8_ on the cross-section at 3.24 and 9.72 Å from the set (0 1 0) crystal plane, respectively.

The degree of electron localization at different locations in a three-dimensional real space can be expressed by the ELF, with a value ranging from 0 to 1, which quantitatively characterizes the degree of electron localization. A closer ELF value to 1 corresponds to a stronger localization of electrons in this region and greater difficulty for electrons to escape. Issues, such as charge-transfer bonds, metallic bonds, and hydrogen bonds, have been extensively studied using ELF [41]. Figure 12 shows the three-dimensional ELF diagrams of SrAl_2_Si_2_O_8_ obtained via VESTA as well as the two-dimensional ELF diagrams in sections of 3.24 and 9.72 Å away from the setting plane in the direction of the (0 1 0) crystal plane. The ELF values near the Sr and O atoms were relatively high, indicating that the localization of electrons near the two atoms was relatively strong.

**Figure 13:**
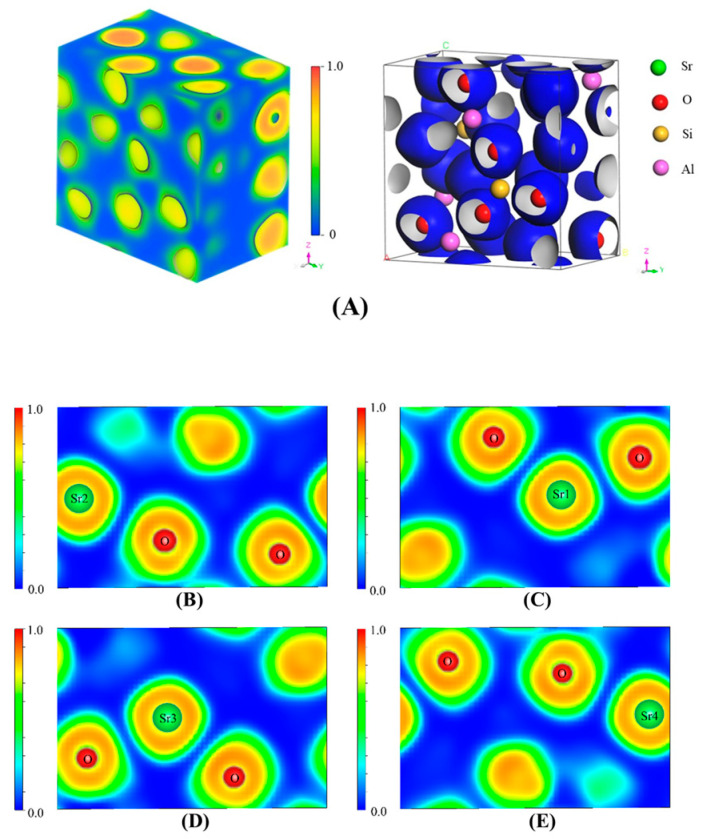
(**A**) Three-dimensional ELF diagrams of Sr_2_Al_2_SiO_7_ (**B**–**E**) are two-dimensional electron localization diagrams of Sr_2_Al_2_SiO_7_ on the cross-section at 1.175, 2.74, 5.09, and 6.66 Å from the set (1 0 0) crystal plane, respectively.

Moreover, the Sr atoms were contracted at the side that was neighboring the O atoms, and the same contraction of ELF was also observed at the O atom in Figure 12B,C [42]. Thus, Sr and O were most likely to combine with each other in the form of ionic bonds. Figure 13A shows the three-dimensional ELF diagrams of Sr_2_Al_2_SiO_7_, and the four Sr atoms in the crystal lattice are marked in Figure 13B–E. Similar to SrAl_2_Si_2_O_8_, the localization of electrons around the Sr and O atoms was relatively high. Therefore, we inferred that Sr and O also combined with each other in the form of ionic bonds in the Sr_2_Al_2_SiO_7_ crystal.

### 3.6. Comparison of Different Sr-Containing Ceramic Solidified Products

**Figure 14:**
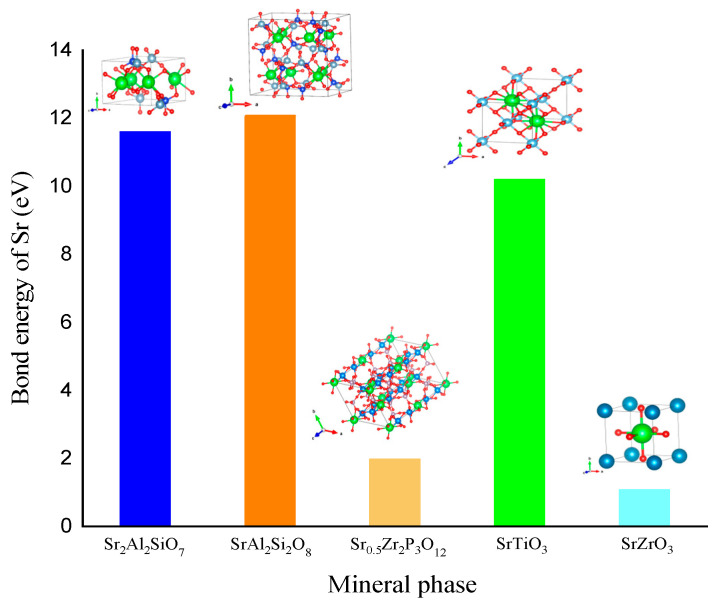
Bond energies of Sr in different mineral phases.

**Figure 15:**
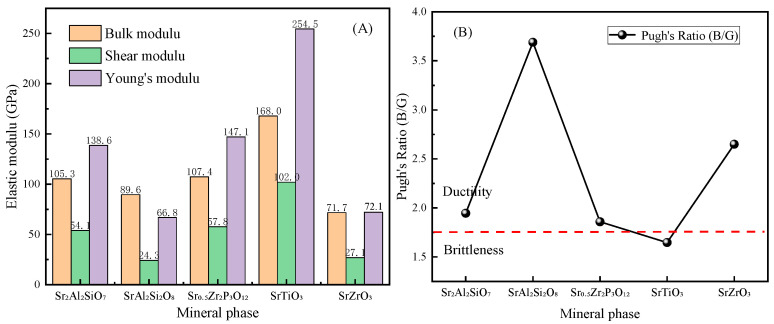
(**A**) Elastic modulus and (**B**) Pugh’s ratios of different mineral phases.

The selection of mineral substrates is currently the primary issue in the preparation and research of Sr-containing solidified products. In this study, three typical Sr-containing ceramic solidified products, Sr_0_._5_Zr_2_(PO_4_)_3_, SrTiO_3_, and SrZrO_3_, were selected, and the bond energy and mechanical properties of Sr in different mineral phases were calculated using VASP and compared with those of SrAl_2_Si_2_O_8_ and Sr_2_Al_2_SiO_7_. Then, the chemical stabilities and mechanical properties of the solidified products were evaluated. The Sr bond energies in the different mineral phases are shown in Figure 14, and the elastic moduli of the different mineral phases are shown in Figure 15A. The Sr bond energies of SrAl_2_Si_2_O_8_ and Sr_2_Al_2_SiO_7_ were higher than those of the other three mineral phases, i.e., 11.6 and 12.1 eV, respectively. This order of magnitude is higher than that of Sr_0_._5_Zr_2_(PO_4_)_3_ with apatite structures and SrZrO_3_ with zirconium-based perovskite structures. This finding indicates good chemical stability in the solidified product containing Sr produced in this study.

The bulk modulus B, shear modulus G, and Young’s modulus E of several Sr-containing mineral phases all exhibited the following trend: SrTiO_3_ > Sr_0_._5_Zr_2_(PO_4_)_3_ > Sr_2_Al_2_SiO_7_ > SrAl_2_Si_2_O_8_ > SrZrO_3_. Among these mineral phases, SrTiO_3_ exhibited the best mechanical properties, whereas SrZrO_3_ exhibited the worst. The proportions of Sr_2_Al_2_SiO_7_, SrAl_2_Si_2_O_8_, and mullite in the solidified product changed continuously with increased Sr content. The formation of the mullite crystal phase can lead to enhanced hardness and modulus in the solidified product [28]. Therefore, a decrease in the mullite proportion may be the main reason for the decline in the mechanical properties of the solidified product. Therefore, to increase the mechanical properties of the solidified product, the content of allophane during the immobilization of Sr should be appropriately increased. The Pugh’s ratio in Figure 15B also shows that only SrTiO_3_ had partial brittleness, and the other ceramic solidified products had a certain ductility.

## 4. Conclusions

The immobilization performance of the natural mineral allophane on the heat-generated nuclide strontium was investigated through ceramic solidification. Stable crystals called Sr_2_Al_2_SiO_7_ and SrAl_2_Si_2_O_8_ were formed in the solidified products after sintering at 1200 °C. These crystalline materials enabled the effective immobilization of strontium, and the immobilization ratio reached 100%. DFT simulations further revealed the structural characteristics and stabilities of the sintered products. The increased strontium content caused a transition from brittle to ductile crystal. From the ELF diagrams, we inferred that Sr and O are highly likely to be bonded to each other through ionic bonds in the two crystals mentioned above. Moreover, the Sr bond energies of SrAl_2_Si_2_O_8_ and Sr_2_Al_2_SiO_7_ were 11.6 and 12.1 eV, respectively, higher than those of the other three common mineral phases. These results indicate that the natural mineral allophane is promising for the final disposal of strontium.

## Figures and Tables

**Table 1:** The cell parameters of SrAl_2_Si_2_O_8_ and Sr_2_Al_2_SiO_7_ after optimization.

Crystal	Crystal System	Space Group	Lattice Parameter					
			a (Å)	b (Å)	c (Å)	α	β	γ
SrAl_2_Si_2_O_8_	Monoclinic	C2/c	9.385	9.385	9.650	81.056°	81.056°	74.481°
Sr_2_Al_2_SiO_7_	Triclinic	P1	7.837	5.264	7.848	90.231°	90.000°	90.000°

**Table 2:** The stiffness tensor parameters of SrAl_2_Si_2_O_8_ and Sr_2_Al_2_SiO_7_ after optimization.

Crystal	Stiffness Tensor *C_ij_*/GPa						
SrAl_2_Si_2_O_8_	*C*_11_	*C*_12_	*C*_13_	*C*_15_	*C*_22_	*C*_23_	*C*_25_
	154.742	43.414	72.793	13.905	187.346	52.2	−16.89
	*C*_33_	*C*_35_	*C*_44_	*C*_46_	*C*_55_	*C*_66_	
	129.94	−0.01	14.429	9.931	45.872	13.305	
Sr_2_Al_2_SiO_7_	*C*_11_	*C*_12_	*C*_13_	*C*_14_	*C*_15_	*C*_16_	*C*_22_
	193.271	63.599	78.3	1.878	0	0	160.477
	*C*_23_	*C*_24_	*C*_25_	*C*_26_	*C*_33_	*C*_34_	*C*_35_
	62.674	0.001	0	0	191.154	−1.141	0
	*C*_36_	*C*_44_	*C*_45_	*C*_46_	*C*_55_	*C*_56_	*C*_66_
	0	46.079	0	0	68.894	−0.546	45.721

## Data Availability

Data are available from the corresponding author on request.
